# Supplementary material for: Variability in Anthocyanin Expression in Native Maize: Purple Totomoxtle as a Phenotypic Trait of Agroecological Value
Source: Plants (Basel). 2025 Aug 12;14(16):2511. doi: 10.3390/plants14162511 (PMC12389302; doi:10.3390/plants14162511)
Supplement: Supplementary file 1 [file plants-14-02511-s001.zip › plants-3792356-supplementary.pdf]

**Table S1.** Photosynthetic pigments in vegetative stages of sibling-cross parents and their inter-population crosses of native maize.

| Pedigree | Chlorophyll-A | Chlorophyll-B | Total chlorophyll | Carotenoids |
|---|---|---|---|---|
| | mg mL$^{-1}$ | | | |
| MCxMor | 56.37$^{A}$ | 21.41$^{A}$ | 77.79$^{A}$ | 21.73$^{AB}$ |
| MC# | 50.99$^{AB}$ | 19.17$^{A}$ | 70.15$^{AB}$ | 21.54$^{AB}$ |
| LLMJxJCTM | 50.45$^{AB}$ | 18.63$^{A}$ | 69.08$^{AB}$ | 21.67$^{AB}$ |
| Mor# | 45.75$^{AB}$ | 14.85$^{ABC}$ | 60.60$^{AB}$ | 20.03$^{AB}$ |
| MorxMC | 45.44$^{AB}$ | 18.01$^{AB}$ | 63.45$^{AB}$ | 22.83$^{A}$ |
| JCTMxRP | 41.72$^{ABC}$ | 17.67$^{AB}$ | 59.39$^{AB}$ | 20.51$^{AB}$ |
| JCTMxLLMJ | 38.06$^{BC}$ | 10.82$^{BC}$ | 48.88$^{BC}$ | 14.91$^{BC}$ |
| LLMJ# | 28.43$^{C}$ | 8.67$^{C}$ | 37.10$^{C}$ | 9.15$^{C}$ |
| JCTM# | 26.96$^{C}$ | 10.76$^{BC}$ | 37.72$^{C}$ | 11.98$^{C}$ |
| MCTM# | 26.06$^{C}$ | 9.17$^{C}$ | 35.23$^{C}$ | 11.79$^{C}$ |
| $\bar{X}$ | 41.02 | 14.91 | 55.93 | 17.61 |
| $p$ | 0.0001 | 0.0001 | 0.0001 | 0.0001 |
| Day/Phenology | | | | |
| Day 19 | 31.19$^{B}$ | 16.76$^{B}$ | 47.95$^{B}$ | 22.67$^{A}$ |
| Day 43 | 65.63$^{A}$ | 20.28$^{A}$ | 85.91$^{A}$ | 21.93$^{A}$ |
| Day 65 | 26.24$^{B}$ | 7.70$^{C}$ | 33.95$^{C}$ | 8.24$^{B}$ |
| HSD= $_{0.05}$ | 6.43 | 2.99 | 8.68 | 3.08 |

The means followed by a different letter within column are significantly different at $p < 0.05$.
